# Supplementary material for: RUBICON: a framework for designing efficient deep learning-based genomic basecallers
Source: Genome Biol. 2024 Feb 16;25:49. doi: 10.1186/s13059-024-03181-2 (PMC10870431; doi:10.1186/s13059-024-03181-2)
Supplement: Supplementary file 1 — Additional file 1. Supplementary notes S1-S6, Figs. S1-S9, and Tables S1-S3. [file 13059_2024_3181_MOESM1_ESM.pdf]

Supplementary Material for RUBICON: a framework for designing efficient deep learning-based genomic basecallers

S1. Quantization-aware basecaller architecture search (QABAS)

QABAS automates the process of finding efficient and high-performance hardware-aware genomics basecallers. Additional file 1: Figure S1 shows the workflow overview of QABAS. The raw sequencing data ⓐ is provided as input to QABAS, which can be obtained through sequencing a new sample, downloading from publicly-available databases, or computer simulation. QABAS uses such a set of data as training ($\mathbb{D}_{train}$) and evaluation set ($\mathbb{D}_{eval}$) while automatically designing a basecaller. To achieve a basecaller design that provides high throughput, we add hardware constraints ⓑ, in terms of latency or throughput, to QABAS. A hardware-aware basecaller can better use the underlying hardware features and greatly accelerate inference speed. As a result, it improves the overall basecalling efficiency.

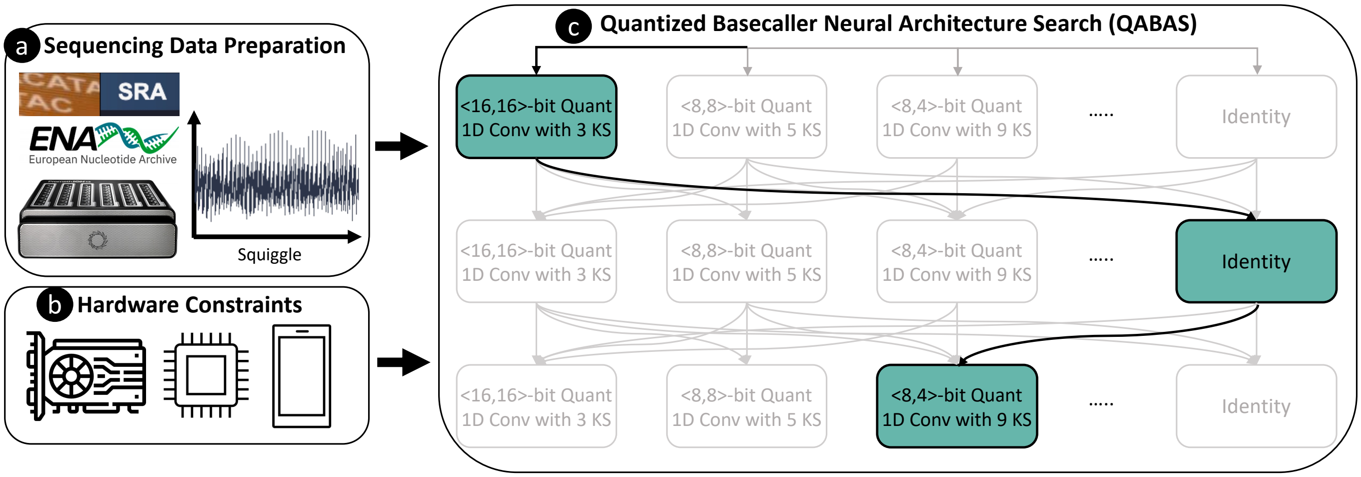

Figure S1: Overview of QABAS. QABAS evaluates a different set of candidate operations for convolution (conv) and quantization bits. In the figure, we show different options for kernel size (KS) (e.g., 3, 5, 9, etc.) and quantization bits (4-b, 8-b, and 16-b) for each network layer. The identity operator removes a layer to get a shallower network.

QABAS ⓒ leverages automated machine learning (AutoML) algorithms [57] using neural architecture search (NAS) to design an efficient hardware basecaller by exploring and evaluating different neural network architectures from a pre-defined search space. The search space $\mathcal{M}$ consists of the possible neural network architectural options while $\mathbb{M} \in \mathcal{M}$ is a sub-architecture from $\mathbb{M}$. The goal is to find an optimal sub-architecture $\mathbb{M}^*$ using Equation S1 that minimizes the training loss ($\mathcal{L}_{train}$) while going over $\mathbb{D}_{train}$ and gives maximum accuracy with the $\mathbb{D}_{eval}$.

$$\mathbb{M}^* = \arg\max_{\mathbb{M}\in\mathcal{M}} Eval(\mathbb{M}, \arg\min_{w^*} \mathcal{L}_{train}(w^*(\mathbb{M}), \mathbb{D}_{train}); \mathbb{D}_{eval}) \quad \text{(S1)}$$

where $w^*(\mathbb{M})$ represent the weights of sub-architecture $\mathbb{M}^*$.

QABAS search space. We define the search space $\mathcal{M}$ as sufficiently large to enable a powerful neural architecture search. A larger space enables the search algorithm to cover more architectures to increase the chance of finding a powerful architecture. However, a larger search space makes converging more difficult for the search algorithm.

Our model search space has sequentially connected blocks, where each block receives input from its direct previous block. We formulate the NAS problem for hardware-aware genomics basecaller as finding: (a) the computational operations in each basic block[3] of a basecaller, including operations in a skip connection block, and (b) quantization bit-width for weights and activations for each neural network layer to perform low-precision computation. Quantization is the reduction of the bit-width precision at which calculations are performed in a neural network to reduce memory and computational complexity. Adding quantization exploration dramatically increases the model search space ($\sim$6.72$\times 10^{20}$ additional viable options in our search space). However, performing a joint search for computational blocks and quantization bits is crucial because: (1) optimizing these two components in separate stages could lead to sub-optimal results as the best network architecture for the full-precision model is not necessarily the optimal one after quantization, and (2) independent exploration would also require considerable search time and energy consumption because of many viable design options [124]. Therefore, `QABAS` searches for both the computational operations present in each basic block of a basecaller and the quantization bits used by these computational operations. In doing so, we tailor the neural network architecture and computation to align with the hardware's capabilities.

`QABAS` search algorithm. `QABAS` evaluates different neural network architectures using differentiable neural architecture search (DNAS) [125–127]. DNAS follows a weight-sharing approach of reusing weights of previously optimized architectures from the neural architecture search space. For example, if sub-architecture $\mathbb{M}_1$ has only one additional layer compared to sub-architecture $\mathbb{M}_2$. In such a scenario, $\mathbb{M}_1$ can use most weights from $\mathbb{M}_2$. Therefore, the search procedure gets accelerated in DNAS compared to training each sub-architecture individually.

DNAS formulates the entire search space as a super-network and distills a target network from this super-network. Traditional NAS approaches [57] often sample many different architectures from the search space and train each architecture from scratch to validate its performance. Such an approach requires heavy computational resources that could lead to thousands of GPU hours of overhead. One way to overcome this issue is to use NAS with heuristic-based methods [128, 129], such as genetic algorithms that select individual architectures from the current *population* to be *parents* and uses them to produce the *children* for the next generation. However, such methods still suffer from the problem of retraining each sample architecture from scratch. Therefore, DNAS provides an efficient solution by sharing computation among different architectures, as many of them have similar properties.

In `QABAS`, we construct an over-parameterized super-network with all possible candidate options. The super-network shares weights among sub-architecture. During the search phase, `QABAS` searches for the optimal: (a) architectural parameter α: likelihood that a computational operation will be preserved in the final architecture; and (b) network weights w: weights of convolution layers. We use ProxylessNAS [130] to binarize the architectural parameter (i.e., $\alpha \in \{0,1\}$) to reduce memory consumption during the search phase. At the end of the search phase, the operators with the highest architectural weight are preserved, while others are eliminated. Since the NAS search procedure is focused on optimizing the super-network, the final sub-network architecture $\mathbb{M}^*$, with all the preserved operations, is retrained to convergence to fully optimize its network weights.

Quantization-aware hardware metric. Current state-of-the-art basecallers [29, 30, 40–43, 45, 73] are hardware-agnostic. They only focus on improving the accuracy without paying attention to its inference efficiency. For example, Fast-bonito [42] uses NAS for basecalling architecture search, however, it does not consider any hardware-related metrics during the architecture search. Therefore, such approaches lead to

[3]Our basic block consists of one-dimensional (1-D) convolution, batch normalization [122], and rectified linear unit (ReLU) [123].

over-provisioned basecallers with a large number of parameters and model sizes that are unoptimized for mixed-precision computation (see Section 2.1). We overcome this inefficiency in `QABAS` by adding hardware constraints, in terms of inference latency, to the `QABAS` search phase. Thus, `QABAS` aims to find an efficient neural network architecture for basecalling that is also optimized for hardware implementation. During the search process, `QABAS` sequentially selects a sub-network from the super-network. The expected latency of the sub-network is the sum of the latencies of each operation in the network. Before the start of the `QABAS` search phase, we profile the latencies of operations present in the search space on targeted hardware to build a latency estimator. We also incorporate the latency while using different quantization bit-widths for the weights and activations in our latency estimator. This latency estimator is utilized to guide the `QABAS` search process.

`QABAS`'s objective function ($\mathcal{L}_{\texttt{QABAS}}$) minimizes a joint cross-entropy error to: (a) provide better basecalling accuracy by minimizing the training loss ($\mathcal{L}_{train}$) while going over $\mathbb{D}_{train}$, and (b) minimize a regularization term ($\mathcal{L}_{reg}$) to find a sub-network $\mathbb{M}$ with inference latency ($\mathbb{L}_{\mathbb{M}}$) that satisfies our inference latency constraints. We add latency constraints by using a target latency parameter ($\mathbb{L}_{tar}$) to the regularization term $\mathcal{L}_{reg}$ to guide the search process. For example, in case we want a small model, then we can provide a higher $\mathbb{L}_{tar}$ value, or vice versa.

$$\mathcal{L}_{\texttt{QABAS}} = \mathcal{L}_{train} + \lambda \mathcal{L}_{reg}$$
$$\mathcal{L}_{reg} = (\mathbb{L}_{\mathbb{M}} - \mathbb{L}_{tar})/\mathbb{L}_{tar}$$

where λ is a parameter to control the tradeoff between the basecalling accuracy and the model latency. As different hardware provides different latencies for the same layers chosen from the `QABAS` search space, the user can customize the `RUBICON` framework for their target hardware by adjusting hardware-specific parameters (i.e., using the `applied_hardware` flag in `RUBICON` [119]). We provide an additional `reference_latency` flag in `QABAS` to guide the search of basecalling architecture to find an architecture that meets certain latency constraints. This coupling of target hardware latency ensures the basecaller architecture is finely tuned to operate optimally on the intended hardware. We provide an example latency estimator for our target hardware (i.e., AIE) in `RUBICON`. However, our integration with the open-source nn-Meter [99] tool allows users to freely configure hardware settings through the `applied_hardware` flag in `RUBICON`. This integration enhances adaptability, enabling efficient optimization and deployment across different hardware environments.

S2. `SkipClip`: Skip connection removal by teaching

Deep neural networks often rely on skip connections to address vanishing gradient problems during training [53]. Skip connections provide a direct path for error propagation, allowing gradients to flow without vanishing [131]. Additionally, they prevent saturation issues in deep neural networks, making them more effective. Similarly, deep learning-based basecallers [29, 30, 40–43, 45, 73] use skip connections to mitigate the vanishing gradient and saturation problems. However, adding skip connections introduces the following three issues for hardware acceleration. First, skip connections increases the data-lifetime. The layers whose activations are reused in subsequent layers must wait for this activation reuse (or buffer the activations in memory) before accepting new input and continuing to compute. This leads to high resource and storage requirements due to data duplication. Second, they introduce irregularity in neural network architecture as these connections span across non-adjacent layers. Third, skip connections require additional computation to adjust the channel size to match the channel size at the non-consecutive layer's input. Thus, increasing model parameters and model size. Therefore, networks without skip connections have more regular topologies that translate better to hardware acceleration.

To address these issues, we propose SkipClip, a first skip connection remover for basecallers. SkipClip gradually removes skip connections using knowledge distillation (KD) [58, 64], where a pretrained larger model (teacher) guides a smaller model (student) to maintain performance without skip connections. As shown in Additional file 1: Figure S2, SkipClip starts with a pretrained over-parameterized model as the teacher, which is not updated during the training of the student network. We use our final QABAS model as the student network. We achieve skip removal by letting the teacher teach the student to perform well on basecalling. At the start of every training epoch, SkipClip removes a skip connection from a block, starting from the input side, while performing KD. This is done until all skip connections are removed from the student network. SkipClip gets the best of both worlds: a highly accurate and topologically regular neural network without skip connections.

During the SkipClip, we perform a forward pass of both the student and the teacher model, while we perform a backward pass only for the student model to update its weights. The loss to update the student network's weight during the backward pass ($\mathcal{L}_{\texttt{SkipClip}}$) is calculated with Equation S2, where we use a weighing of the actual student loss ($\mathcal{L}_S$) and distillation loss ($\mathcal{L}_D$) using an alpha (α) hyper-parameter. The student and the teacher model compute probabilities f_T and f_S for output labels (i.e., nucleotides A, C, G, T) in the forward pass, respectively. We use cross entropy ($\mathcal{L}_{CR}$) in the probability distributions to calculate the distillation loss ($\mathcal{L}_D$) as in Equation S3. The temperature (τ) variable is used for *softening* the probability distributions, i.e., it controls the weight of knowledge from the teacher network for a student network to absorb. As we raise the τ, the resulting soft label probability distribution becomes richer in information.

$$\mathcal{L}_{\texttt{SkipClip}} = \alpha \mathcal{L}_S - (1 - \alpha)\mathcal{L}_D \quad \text{(S2)}$$

$$where \mathcal{L}_D = \mathcal{L}_{CR}(f_T/\tau, f_S/\tau) \quad \text{(S3)}$$

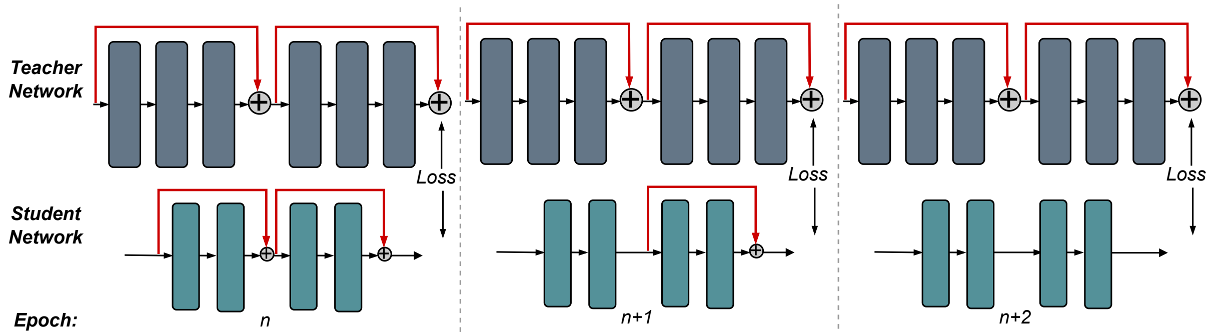

Figure S2: Overview of SkipClip process for three epochs. We start with a large, overprovisioned floating-point precision model as the teacher network and our QABAS mixed-precision model as the student network. During the training, SkipClip removes a skip connection from the student network every n epoch, starting with the first skip connection encountered in the network from the input.

S1. Sensitivity to skip connection

Many state-of-the-art deep learning-based basecallers [29, 30, 40–43, 45, 73] incorporate skip connections to improve their basecalling accuracy. Additional file 1: Figure S3 shows the accuracy of Bonito_CTC using two different configurations of skip connections (s1 and s2) and one configuration without any skip connections (s3) and compares it to the baseline Bonito_CTC architecture. In s1 configuration, we reduce the number of repeats in each block to one, while in s2 configuration, we use only one block with maximum channel size, maximum kernel size, and the maximum number of repeats.

For s3 configuration, we manually remove all the skip connections from each block in Bonito_CTC. We also annotate the change in model parameters compared to the baseline model. Bonito_CTC architecture

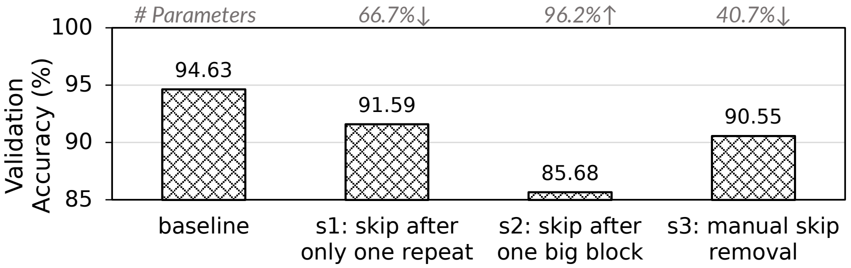

Figure S3: Basecaller sensitivity to skip connections.

comprises several blocks, each consisting of a time channel separable convolution sub-block (referred to as repeat). We make two major observations. First, the number of sub-blocks we provide skip connection plays an important role. In `s1` configuration, we observe that by using only one repeat, we reduce the accuracy by 2.84% with 66.7% lower model parameters, while by merging all the blocks into one big block in `s2` configuration, we observe 8.75% lower accuracy with 96.2% higher model parameters. Second, manually removing all the skip connections in `s3` configuration leads to 40.7% lower model parameters at the expense of a 3.88% loss in accuracy. This performance degradation is because, during neural network training, these connections provide a direct path for propagating the error through the layers and dealing with the vanishing gradient problem, allowing deep networks to learn properly and converge during training. Therefore, manual removal of skip connections can lead to lower basecalling performance. We conclude that skip connections are critical for basecalling accuracy.

S2. Hyper-parameter tuning for `SkipClip`

In Additional file 1: Figure S4, we show the effect of two critical hyper-parameters of `SkipClip` (alpha (α) and temperature (τ)) on the validation accuracy of `Bonito_CTC`. We observe that as we raise α while keeping τ constant, the basecaller accuracy increases. At higher α, `SkipClip` gives more importance to the student loss than the distillation loss during the backward pass. We use α =0.9 throughout our experiments.

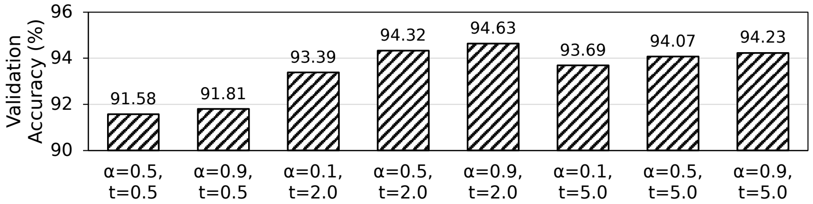

Figure S4: Sensitivity of `SkipClip` to hyper-parameters alpha (α) and temperature (τ).

For τ, we experiment with values ranging from 0.5 to 5.0. Increasing τ provides more knowledge from the teacher network for a student network to absorb. We observe at τ=2, `SkipClip` provides the highest accuracy. Further increasing τ does not provide benefits because the student network cannot absorb knowledge provided by the teacher network.

S3. `RUBICALL` architecture

Additional file 1: Figure S5 shows the architecture of `RUBICALL`. We develop `RUBICALL` using `QABAS` and `SkipClip`. The `RUBICALL` architecture is composed of 28 quantized convolution blocks containing ∼3.3 million model parameters. Each block consists of quantized grouped 1-dimensional convolution and quantized pointwise 1-dimensional convolution where every layer is quantized to a different domain. The convolution operation is followed by batch normalization (Batch Norm) [122] and a quantized rectified linear unit (QuantReLU) [123] activation function. The final output is passed through a connectionist

temporal classification (CTC) [132] layer to produce the decoded sequence of nucleotides (A, C, G, T). CTC is used to provide the correct alignment between the input and the output sequence.

In a learning task, $\mathcal{X}$ represents feature space with label $\mathcal{Y}$, where a machine learning model is responsible for estimating a function $f\colon \mathcal{X} \rightarrow \mathcal{Y}$. RUBICALL first splits a long read in electrical-signal format (e.g., millions of signals) into multiple smaller chunks (e.g., thousands of samples per chunk) and then basecalls these chunks. RUBICALL uses the input signal (or squiggle) as $\mathcal{X}$ to predict nucleotides as label $\mathcal{Y}$. The CTC layer assigns a probability for all possible labels in $\mathcal{Y}$ given an $\mathcal{X}$ at each time-step. The nucleotide with the highest probability is selected as the final output.

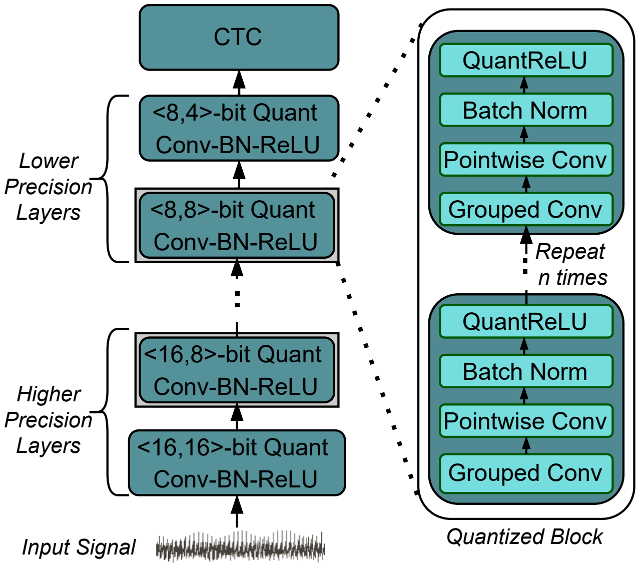

Figure S5: Overview of RUBICALL architecture. The normalized input signal is passed through a succession of quantized convolution blocks. Each block is composed of several processing steps (convolution, batch normalization, and activation). We represent the quantization as a tuple <weight, activation>. Initial layers use a higher precision for weights and activations, while the final layers use a lower precision. The final output is passed through a connectionist temporal classification (CTC) to produce the decoded sequence of nucleotides.

S4. Comparison to more accurate basecallers

Our goal is to make basecalling highly efficient and fast by building the first framework for specializing and optimizing machine learning-based basecaller. Currently, we focus on CNN-based basecallers because: (1) they are the most widely used basecallers, and (2) the fundamental multiply-accumulate (MAC) operation in a CNN model is amenable to hardware acceleration, unlike the operations in RNN-based basecallers. As Bonito_CTC has the same backend as RUBICALL (i.e., Quartznet [50]), we consider it as an expert-designed model. Bonito_CRF's super high accuracy (Bonito_CRF-sup) model is an RNN-based basecaller that provides more accuracy than Bonito_CRF-fast at the expense of a much larger model. We compare the overall basecalling throughput of RUBICALL with that of the baseline basecallers in terms of basecalling accuracy, model parameters, and model size in Additional file 1: Figure S6(a), S6(b), and S6(c), respectively.

In addition to our previous observations from Figure 5, we make three new observations from Additional file 1: Figure S6 and Additional file 1: Table S1. First, RUBICALL-MP has 185.54× the performance of the highly-accurate Bonito_CRF-sup. RUBICALL-MP is the only basecaller that provides both higher performance and accuracy when compared to all the other evaluated basecallers. Second, Bonito_CRF-sup uses

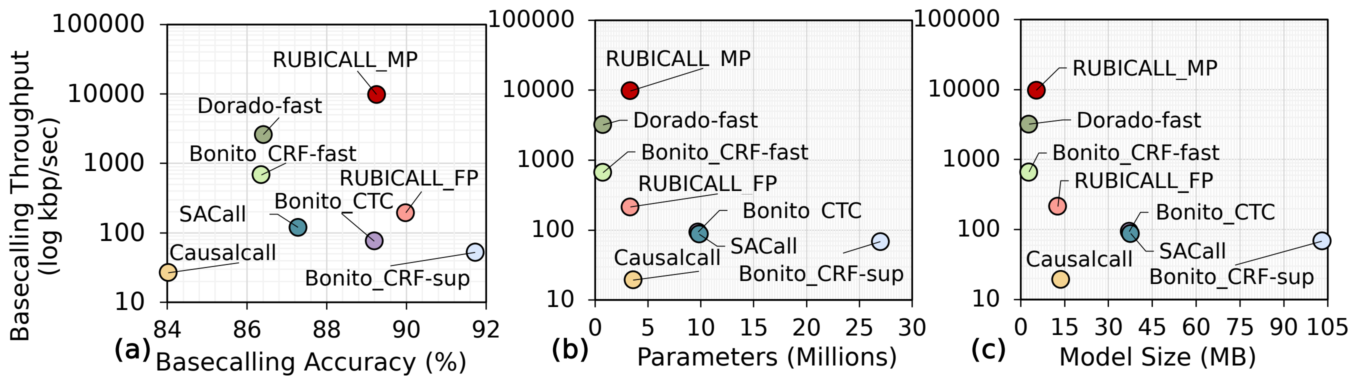

Figure S6: Comparison of average basecalling throughput for `RUBICALL-MP` with baseline basecaller in terms of: (a) average basecalling accuracy, (b) model parameters, and (c) model size.

Table S1: Comparison of `RUBICALL-MP` with baseline basecallers in terms of model architecture, characteristics, precision, basecalling throughput, basecalling accuracy, parameters, and model size. For basecalling throughput and basecalling accuracy, we report average (Avg.), minimum (Min.), maximum (Max.), 25th percentile (25th %tile), and 75th percentile (75th %tile) values for all the basecallers.

Basecaller	Architecture	Characteristics	Precision	Basecalling Throughput (kbp/sec)					Basecalling Accuracy (%)					Parameters	Model Size (MB)
				**Avg.**	**Min.**	**Max.**	**25th %tile**	**75th %tile**	**Avg.**	**Min.**	**Max.**	**25th %tile**	**75th %tile**	–	–
`Causalcall`	CNN	Low Accuracy	FP32	26.76	11.04	53.94	18.65	30.13	84.02	82.70	86.42	83.57	86.17	3,589,893	13.69
`SACall`	Transformer	Low Accuracy	FP32	119.71	47.33	346.30	86.32	112.58	87.28	86.44	91.44	86.97	89.22	9,854,725	37.59
`Bonito_CTC`	CNN	High Accuracy	FP32	76.22	31.68	219.42	54.64	73.46	89.19	87.99	93.75	88.62	91.15	9,738,573	37.15
`RUBICALL-FP`	CNN	High Accuracy	FP16	194.74	99.8	394.9	129.72	240.34	89.25	86.59	93.64	88.97	91.41	3,314,578	12.64
`Bonito_CRF-sup`	RNN	Highest Accuracy	FP16	52.63	20.22	149.2	35.03	55.56	**91.73**	**90.60**	**95.95**	**91.43**	**93.72**	26,992,744	103.03
`Bonito_CRF-fast`	RNN	Fast Performance	FP16	685.13	261.53	1044.37	421.52	881.84	86.36	82.53	91.39	86.25	88.51	730,344	2.79
`Dorado-fast`	RNN	Fast Performance	FP16	2593.34	1155.06	3927.03	1835.99	3351.95	87.16	82.53	91.39	86.25	88.51	730,344	2.79
`RUBICALL-MP`	CNN	High Accuracy and Fast Performance	Mixed-Precision	**9765.65**	**5309.74**	**12862.73**	**8433.53**	**10892.55**	89.25	86.59	93.64	88.97	91.41	3,314,578	5.36

7.52×, 36.96×, 2.77×, 2.74×, 36.96×, and 8.14× model parameters leading to a model size of 7.53×, 36.93×, 2.77×, and 19.22× compared to `Causalcall`, `Bonito_CRF-fast`, `Bonito_CTC`, `SACall`, `Dorado-fast` and `RUBICALL-MP`, respectively. Third, `Bonito_CRF-sup` is 5.37% more accurate than its throughout-optimized version, `Bonito_CRF-fast`, which provides up to 13.02× higher basecalling performance. We conclude that the high accuracy of a basecaller comes at a substantial cost in terms of lower throughput due to the higher number of model parameters and model size.

S5. Evaluation on other hardware platforms

We also evaluate the performance of `RUBICALL` and all the other basecallers on NVIDIA A40 [70] GPU with 48GiB DRAM and AMD EPYC 7442 [87] 24-Core with 256GiB DRAM. Compared to the AMD MI210 [69], the NVIDIA A40 has a 1.65× higher peak compute performance while maintaining a 2.35× lower peak memory bandwidth.

We make two major observations from Additional file 1: Figure S7. First, `RUBICALL-MP` on AIE consistently outperforms A40 by 502.52×, 14.67×, 104.14×, 111.25×, 45.61×, and 3.19× higher performance compared to `Causalcall`, `Bonito_CRF-fast`, `Bonito_CTC`, `SACall`, `RUBICALL-FP`, and `Dorado-fast`, respectively. Second, for compute-bound basecallers, A40 provides 1.23×, 1.18×, and 1.09× higher performance than AMD MI210 (Figure 6) for `Bonito_CTC`, `Dorado-fast`, and `RUBICALL-FP`, respectively. For memory-bound basecallers, A40 provides 1.38×, 1.03×, and 1.36× lower performance for `Causalcall`, `Bonito_CRF-fast`, and `SACall`, respectively. We conclude that `RUBICON` provides benefits across multiple hardware platforms.

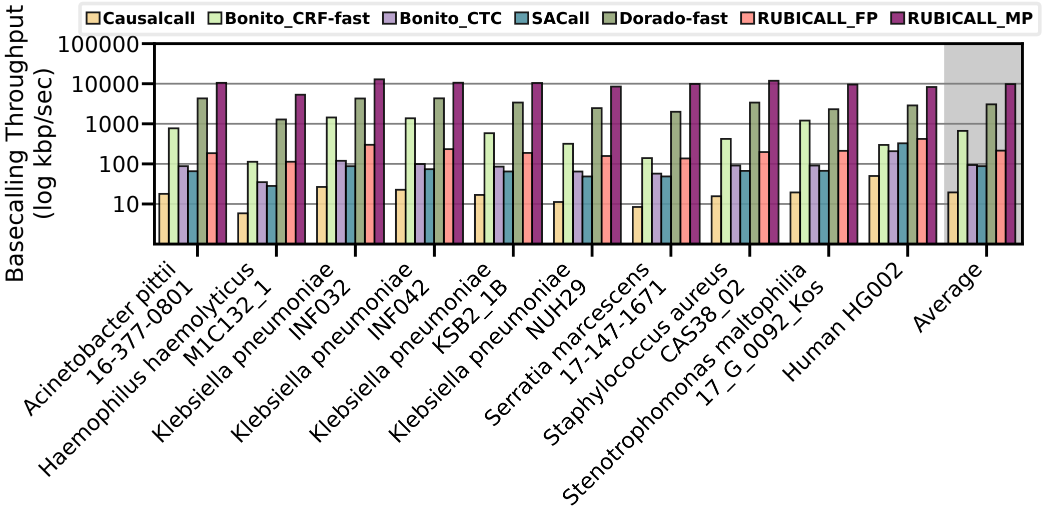

Figure S7: Performance comparison of `RUBICALL` (using floating-point precision (`RUBICALL-FP`) and mixed-precision (`RUBICALL-MP`)) and five state-of-the-art basecallers on NVIDIA A40 [70]. The y-axis is on a logarithmic scale.

S6. Analysis of mapped reads and mapped bases

Additional file 1: Table S2 shows the average read length, the overall number of mapped reads, the number of mapped bases, and the ratio of mapped bases to the mapped reads. Our goal is to evaluate the tools in terms of the read lengths they can generate and the alignable fraction of these reads to their corresponding reference genomes. We make three key observations. First, we find that the average read lengths are similar across different basecallers for each dataset, except `Causalcall` for the human genome. This indicates that the substantial differences in read length are unlikely to influence the ratio of mapped bases to the number of mapped reads, while the number of alignable sequences within each read and the number of mapped reads can have the main effect on such a ratio. Second, we find that basecallers provide a similar number of mapped reads and the ratio of mapped bases to the mapped reads for each dataset, except `Causalcall` for the human genome. These similarities mainly indicate that the unalignable reads and the unalignable regions within each read are likely to be similar across basecallers, leading to similar ratios of mapped bases to mapped reads when mapping reads with similar average read lengths. Third, we find that `Causalcall` provides exceptions for the human genome in terms of the average read length and the mapped bases to the mapped reads ratio. This is mainly because `Causalcall` fails to basecall all raw signals for the human genome and provides a subset of basecalled reads that other basecallers generate, leading to inaccurate analysis overall. We conclude that almost all basecallers, except `Causalcall`, generate reads with similar average read lengths and reads with similar alignable regions, although these similarities differ by certain percentages' as we discuss in Section 2.5.2.

Table S2: Read mapping comparison of RUBICALL with baseline basecallers in terms of mean length of individual sequencing reads in a dataset (Avg. Length), the total number of mapped reads (Mapped Reads), the total number of mapped bases (Mapped Bases), and the ratio of total number of mapped reads to mapped bases.

Dataset	Basecaller	Avg. Length	Mapped Reads	Mapped Bases	#Mapped Bases/ #Mapped Reads
Acinetobacter pittii 16-377-0801	causalcall	25,718.6	4,434	114,159,528	25,746.4
	Bonito_CRF-fast	26,151.3	4,452	110,907,740	24,911.9
	Bonito_CTC	24,879.1	4,457	110,183,466	24,721.4
	SACall	25,153.3	4,451	111,997,940	25,162.4
	Dorado-fast	26,151.1	4,452	110,907,740	24,911.9
	RUBICALL	25,000.8	4,452	111,405,897	25,023.8
Haemophilus haemolyticus M1C132_1	causalcall	NA	NA	NA	NA
	Bonito_CRF-fast	9,835.7	6,444	64,816,196	10,058.4
	Bonito_CTC	8,862.8	6,201	73,573,092	11,864.7
	SACall	6,871.1	4,028	43,233,160	10,733.2
	Dorado-fast	9,844.8	6,444	64,816,196	10,058.4
	RUBICALL	7,751.5	6,287	63,415,299	10,086.7
Klebsiella pneumoniae INF032	causalcall	35,781.9	15,150	542,123,428	35,783.7
	Bonito_CRF-fast	36,556.4	15,147	533,045,454	35,191.5
	Bonito_CTC	35,189.1	15,152	519,659,064	34,296.4
	SACall	35,078.5	15,150	531,456,488	35,079.6
	Dorado-fast	36,624.7	15,147	533,045,454	35,191.5
	RUBICALL	35,420.7	15,153	536,724,002	35,420.3
Klebsiella pneumoniae INF042	causalcall	48,483.8	11,236	542,123,428	48,248.8
	Bonito_CRF-fast	49,617.5	11,252	533,045,454	47,373.4
	Bonito_CTC	46,198.4	11,273	519,659,064	46,097.7
	SACall	46,298.3	11,198	531,456,488	47,459.9
	Dorado-fast	49,621.4	11,252	533,045,454	47,373.4
	RUBICALL	46,637.6	11,268	536,724,002	47,632.6
Klebsiella pneumoniae KSB2_1B	causalcall	24,039.6	16,642	401,041,491	24,098.2
	Bonito_CRF-fast	24,723.9	16,744	384,436,100	22,959.6
	Bonito_CTC	22,918.8	16,803	385,157,295	22,921.9
	SACall	22,917.5	16,371	381,266,978	23,289.2
	Dorado-fast	24,728.0	16,744	384,436,100	22,959.6
	RUBICALL	23,141.9	16,783	388,897,351	23,172.1
Klebsiella pneumoniae NUH29	causalcall	16,233.7	14,954	243,112,795	16,257.4
	Bonito_CRF-fast	16,435.7	15,056	229,123,038	15,218.1
	Bonito_CTC	15,182.0	15,152	233,135,041	15,386.4
	SACall	15,536.5	15,088	234,764,649	15,559.7
	Dorado-fast	16,419.0	15,056	229,123,038	15,218.1
	RUBICALL	15,300.8	15,113	231,523,267	15,319.5
Serratia marcescens 17-147-1671	causalcall	8,198.0	12,729	104,864,058	8,238.2
	Bonito_CRF-fast	8,456.2	16,667	133,916,776	8,034.8
	Bonito_CTC	8,024.8	16,715	133,754,055	8,002.0
	SACall	8,167.1	16,665	136,289,479	8,178.2
	Dorado-fast	8,465.1	16,667	133,916,776	8,034.8
	RUBICALL	8,076.5	16,696	134,916,360	8,080.8
Staphylococcus aureus CAS38_02	causalcall	21,425.2	11,038	236,529,129	21,428.6
	Bonito_CRF-fast	21,932.6	11,047	237,020,597	21,455.7
	Bonito_CTC	21,455.7	11,047	232,091,657	21,009.5
	SACall	21,372.6	11,047	236,103,648	21,372.6
	Dorado-fast	21,930.8	11,047	237,020,597	21,455.7
	RUBICALL	21,501.0	11,047	237,521,476	21,501.0
Stenotrophomonas maltophilia 17_G_0092_Kos	causalcall	31,415.3	15,946	501,018,868	31,419.7
	Bonito_CRF-fast	31,736.6	15,959	470,408,299	29,476.1
	Bonito_CTC	29,453.8	15,997	474,913,094	29,687.6
	SACall	30,095.7	15,985	481,168,698	30,101.3
	Dorado-fast	31,727.6	15,959	470,408,299	29,476.1
	RUBICALL	29,676.7	15,980	474,401,853	29,687.2
Human HG002	causalcall	11,201.7	163,984	2,612,902,733	15,933.9
	Bonito_CRF-fast	37,755.9	238,205	10,627,000,000	44,612.8
	Bonito_CTC	35,831.5	243,686	10,212,000,000	41,906.4
	SACall	32,815.7	203,228	9,080,197,989	44,679.9
	Dorado-fast	37,991.9	238,474	10,627,000,000	44,562.5
	RUBICALL	37,141.2	245,373	10,591,000,000	43,162.9

S7. K-mer counting analysis

We analyze the occurrence of k-mer (i.e., substrings of length k) in a given sequence of basecalled reads and their assemblies in Additional file 1: Figure S8 and Additional file 1: Figure S9, respectively. We use BBMap [133] to collect the number of unique k-mers and the frequency of each unique k-mer in a given sequence. During our analysis, we vary the value of k from 15 to 31. Based on our empirical analysis, we set the k value for our evaluated bacterial species to 15, where we observe distinct peaks of unique k-mers. We do not perform k-mer frequency analysis for the human genome due to the low coverage of the human genome in our experiments. We make the following two observations from Additional file 1: Figure S8 and Additional file 1: Figure S9. First, `RUBICALL` has distinct peaks for all the evaluated species, often matching the k-mer composition generated from `Bonito_CTC`. Second, `Bonito_CRF-fast` and `Dorado-fast` generate similar k-mer compositions as they both have the same neural network architecture.

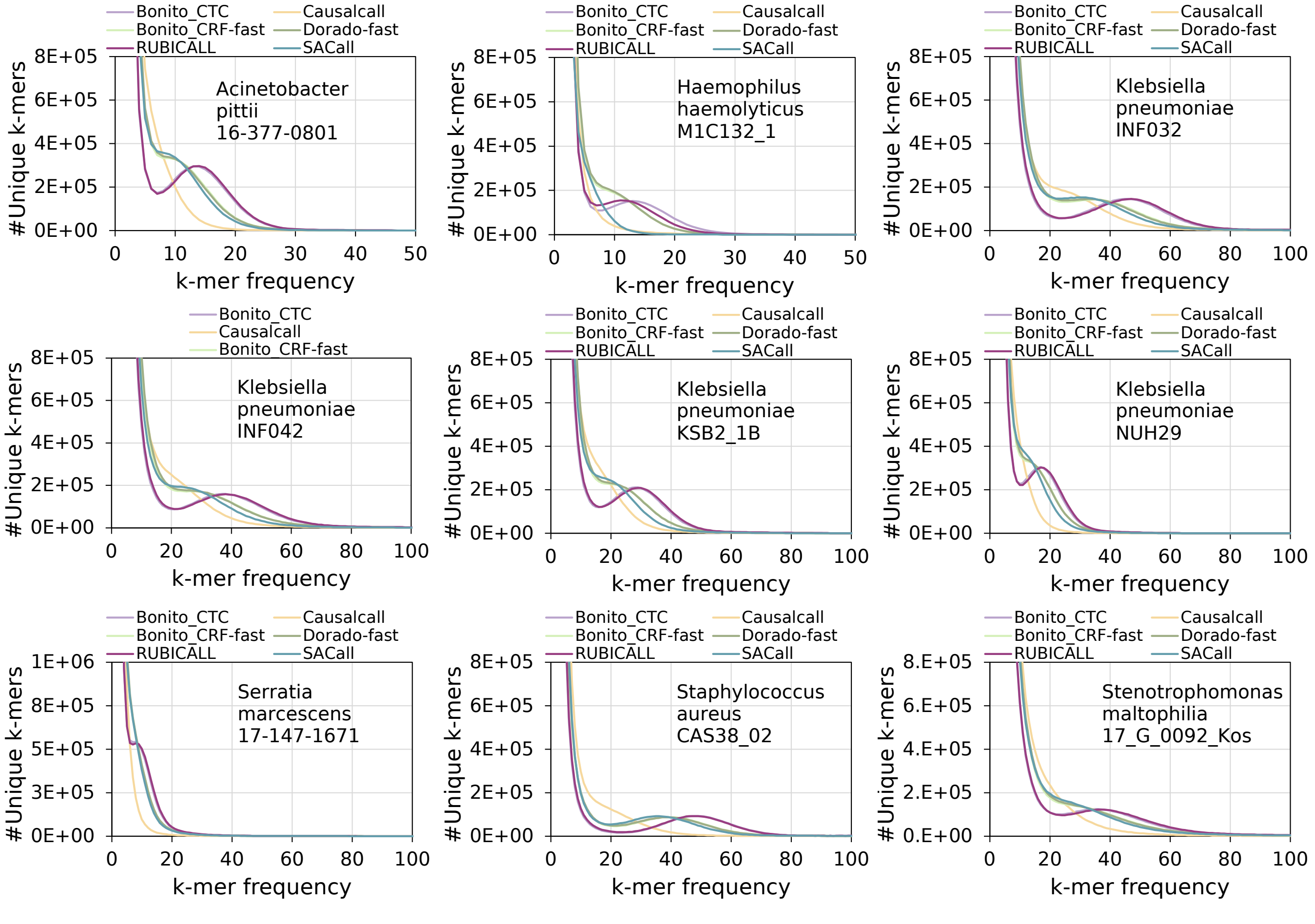

Figure S8: K-mer frequency analysis of generated reads from `RUBICALL` and all the other evaluated basecallers.

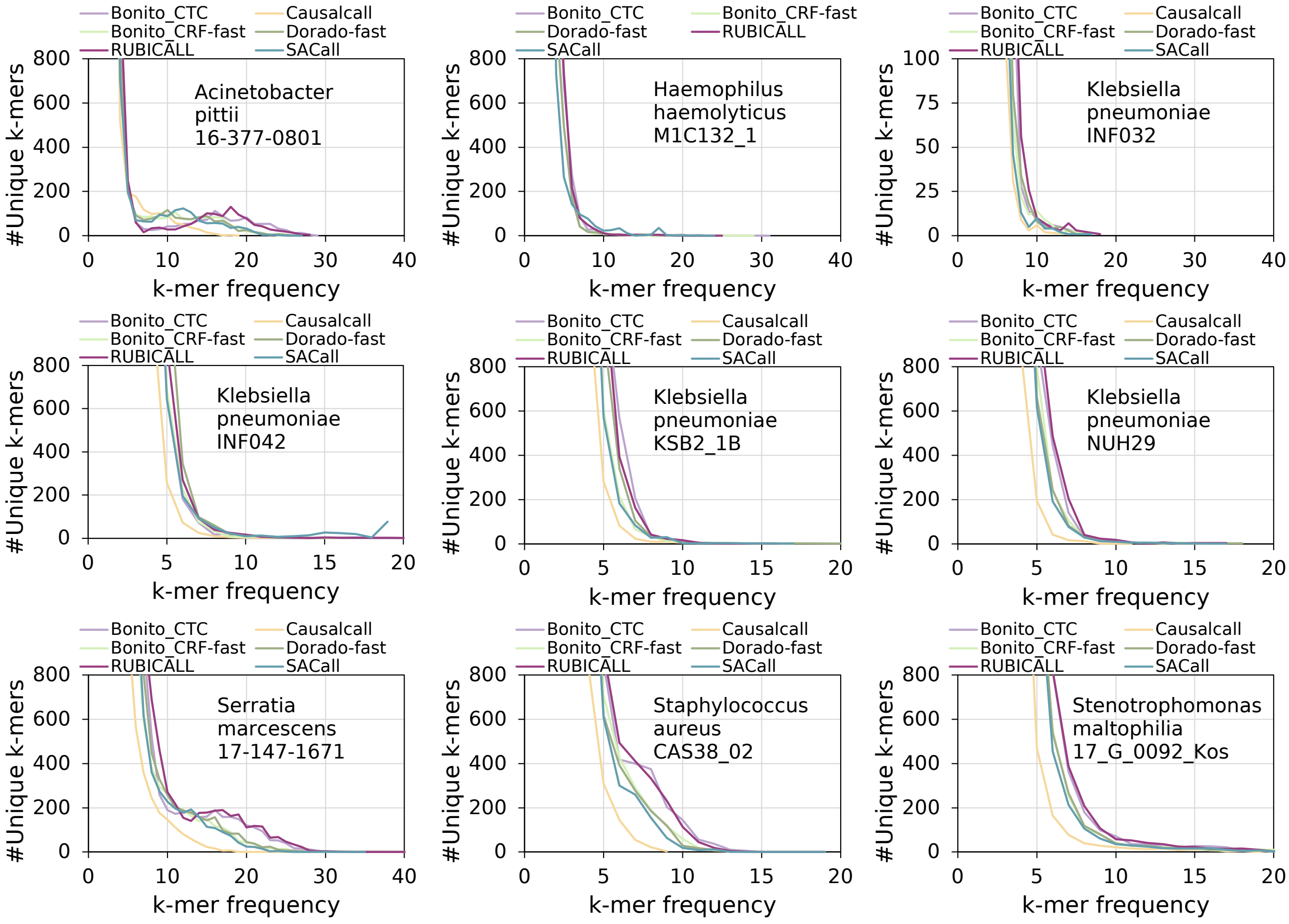

Figure S9: K-mer frequency analysis of generated assemblies of reads from `RUBICALL` and all the other evaluated basecallers.

Additional file 1: Table S3 presents an analysis of k-mer frequencies in the raw reads and the corresponding assemblies. We include common sequences and read-to-assembly ratios to provide a comprehensive view of the similarities and disparities in sequence representation, aiding in assessing data quality and the performance of the assembly algorithms. We observe that the k-mers identified as over-represented in the assemblies are mainly observed as over-represented k-mers in read sets for most basecallers. These over-represented k-mers are likely to appear due to the particular repetitive regions of each genome, making k-mers appear a larger amount of times for these regions. Therefore, there is potentially no additional insertion or depletion of these k-mers during the assembly process.

Table S3: Comparison of under and over-represented sequences (k-mers) in reads and assemblies for all the evaluated basecallers. For both under and over-represented sequences, we show common sequences (`Common`) and the ratio of k-mer frequencies between reads and assemblies (`Ratio`).

		Under-Represented				Over-Represented			
**Dataset**	**Basecaller**	**Read**	**Assembly**	**Common**	**Ratio**	**Read**	**Assembly**	**Common**	**Ratio**
Acinetobacter pittii 16-377-0801	Causalcall	73,096,681	3,768,504	3,221,668	0.855	6,263	187	187	1.000
	Bonito_CRF-fast	55,359,526	3,747,817	2,367,806	0.632	17,983	360	360	1.000
	Bonito_CTC	44,790,782	3,593,419	1,814,762	0.505	29,097	296	296	1.000
	SACall	55,660,535	3,625,236	2,381,232	0.657	15,534	368	368	1.000
	Dorado-fast	55,775,603	3,760,029	2,404,108	0.639	18,137	425	425	1.000
	RUBICALL	44,085,891	3,609,296	1,793,772	0.497	30,316	430	430	1.000
Haemophilus haemolyticus M1C132_1	Causalcall	31,021,572	NA	NA	NA	1,552	NA	NA	NA
	Bonito_CRF-fast	42,355,232	2,077,823	2,076,203	0.999	2,865	54	53	0.981
	Bonito_CTC	35,847,257	1,919,667	700,997	0.365	33,713	94	94	1.000
	SACall	36,998,888	2,000,357	1,998,679	0.999	22,517	98	61	0.622
	Dorado-fast	41,939,332	2,074,317	2,072,740	0.999	4,714	37	37	1.000
	RUBICALL	33,917,316	1,929,302	1,127,668	0.584	23,221	100	100	1.000
Klebsiella pneumoniae INF032	Causalcall	197,327,230	4,850,481	3,833,326	0.790	15,891	6	6	1.000
	Bonito_CRF-fast	169,124,267	4,914,464	3,353,593	0.682	35,175	22	22	1.000
	Bonito_CTC	155,835,445	4,758,242	2,718,894	0.571	53,149	20	20	1.000
	SACall	176,768,581	4,747,776	3,366,011	0.709	28,038	12	12	1.000
	Dorado-fast	167,899,392	4,916,810	3,351,520	0.682	35,059	24	24	1.000
	RUBICALL	150,348,189	4,776,357	2,689,193	0.563	62,356	26	26	1.000
Klebsiella pneumoniae INF042	Causalcall	211,565,073	5,171,081	4,222,032	0.816	22,204	3	3	1.000
	Bonito_CRF-fast	178,074,237	5,212,872	3,532,743	0.678	61,454	29	29	1.000
	Bonito_CTC	162,568,221	4,964,190	2,843,401	0.573	81,948	36	36	1.000
	SACall	186,186,165	5,024,228	3,609,853	0.718	40,440	41	41	1.000
	Dorado-fast	174,755,139	5,508,965	3,808,628	0.691	63,644	23	23	1.000
	RUBICALL	158,433,298	4,989,328	2,818,523	0.565	92,045	37	37	1.000
Klebsiella pneumoniae KSB2_1B	Causalcall	180,267,220	5,064,568	4,648,903	0.918	8,844	6	6	1.000
	Bonito_CRF-fast	152,878,553	5,122,808	4,044,261	0.789	23,755	16	16	1.000
	Bonito_CTC	137,461,268	4,859,560	3,174,990	0.653	27,211	14	14	1.000
	SACall	158,736,471	4,903,831	4,117,531	0.840	16,090	12	12	1.000
	Dorado-fast	150,458,414	5,265,881	4,164,287	0.791	24,938	19	19	1.000
	RUBICALL	134,066,854	4,876,789	3,186,025	0.653	31,451	17	17	1.000
Klebsiella pneumoniae NUH29	Causalcall	140,405,375	5,060,601	5,004,375	0.989	835	1	1	1.000
	Bonito_CRF-fast	110,315,181	5,060,829	4,696,878	0.928	4,320	22	22	1.000
	Bonito_CTC	97,405,757	4,775,503	4,182,794	0.876	5,177	20	20	1.000
	SACall	112,996,097	4,844,709	4,613,301	0.952	2,941	16	16	1.000
	Dorado-fast	108,585,877	5,043,253	4,679,036	0.928	4,483	23	23	1.000
	RUBICALL	95,201,166	4,789,453	4,136,482	0.864	5,645	25	25	1.000
Serratia marcescens 17-147-1671	Causalcall	66,514,376	5,334,807	5,193,034	0.973	30,238	4	4	1.000
	Bonito_CRF-fast	63,963,265	5,399,858	4,554,600	0.843	61,321	4	4	1.000
	Bonito_CTC	53,413,056	5,217,101	3,821,412	0.732	61,275	9	9	1.000
	SACall	64,337,585	5,284,226	4,534,343	0.858	54,872	1	1	1.000
	Dorado-fast	63,535,166	5,451,215	4,583,027	0.841	62,006	4	4	1.000
	RUBICALL	51,724,568	5,243,385	3,741,711	0.714	64,943	9	9	1.000
Staphylococcus aureus CAS38_02	Causalcall	106,477,765	2,791,690	2,784,563	0.997	3,375	3	3	1.000
	Bonito_CRF-fast	72,908,007	2,813,307	2,774,962	0.986	12,170	33	33	1.000
	Bonito_CTC	59,047,673	2,736,120	2,669,644	0.976	18,475	37	37	1.000
	SACall	73,372,106	2,741,321	2,710,663	0.989	10,487	29	29	1.000
	Dorado-fast	73,708,021	2,824,623	2,786,854	0.987	11,902	37	37	1.000
	RUBICALL	58,939,315	2,739,823	2,675,209	0.976	18,186	84	84	1.000
Stenotrophomonas maltophilia 17_G_0092_Kos	Causalcall	183,625,102	4,653,477	4,000,323	0.860	25,103	132	132	1.000
	Bonito_CRF-fast	144,980,026	4,647,752	3,083,896	0.664	127,258	210	210	1.000
	Bonito_CTC	127,334,549	4,383,078	2,495,090	0.569	170,398	285	285	1.000
	SACall	139,640,543	4,422,404	3,045,239	0.689	112,124	201	201	1.000
	Dorado-fast	143,087,662	4,594,794	3,087,855	0.672	127,653	201	201	1.000
	RUBICALL	121,924,168	4,391,308	2,430,608	0.554	197,883	327	327	1.000
